# Dexamethasone for treating SARS-CoV-2 infection: a systematic review and meta-analysis

**DOI:** 10.1590/1516-3180.2021.0120.R1.30062021

**Published:** 2021-10-11

**Authors:** Lirane Elize Defante Ferreto, Durcelina Schiavoni Bortoloti, Paulo Cezar Nunes Fortes, Franciele Follador, Gisele Arruda, João Paulo Ximenez, Guilherme Welter Wendt

**Affiliations:** I PhD. Pharmacist and Associate Professor, Centro de Ciências da Saúde (CCS), Universidade Estadual do Oeste do Paraná (UNIOESTE), Francisco Beltrão (PR), Brazil.; II PhD. Physical Education Professional and Adjunct Professor of Research Methods, Department of Biological Sciences, Medical and Health, Universidade Paranaense (UNIPAR), Umuarama (PR), Brazil.; III MD, PhD. Intensive Care Unit Physician and Adjunct Professor, Centro de Ciências da Saúde (CCS), Universidade Estadual do Oeste do Paraná (UNIOESTE), Francisco Beltrão (PR), Brazil.; IV PhD. Chemist and Associate Professor, Centro de Ciências da Saúde (CCS), Universidade Estadual do Oeste do Paraná (UNIOESTE), Francisco Beltrão (PR), Brazil.; V PhD. Biologist and Adjunct Professor, Centro de Ciências da Saúde (CCS), Universidade Estadual do Oeste do Paraná (UNIOESTE), Francisco Beltrão (PR), Brazil.; VI PhD. Pharmacist and Postdoctoral Researcher, Faculdade de Medicina de Ribeirão Preto (FMRP), Universidade de São Paulo (USP), Ribeirão Preto (SP), Brazil.; VII PhD. Psychologist and Adjunct Professor of Quantitative Research Methods, Centro de Ciências da Saúde (CCS), Universidade Estadual do Oeste do Paraná (UNIOESTE), Francisco Beltrão (PR), Brazil.

**Keywords:** Dexamethasone, COVID-19, SARS-CoV-2, Meta-analysis [publication type], Pulmonary medicine, Coronavirus pandemic, Drug repurposing, Respiratory medicine, Clinical trials

## Abstract

**BACKGROUND:**

Considering the disruptions imposed by lockdowns and social distancing recommendations, coupled with overwhelmed healthcare systems, researchers worldwide have been exploring drug repositioning strategies for treating severe acute respiratory syndrome coronavirus 2 (SARS-CoV-2).

**OBJECTIVE:**

To compile results from randomized clinical trials on the effect of dexamethasone, compared with standard treatment for management of SARS-CoV-2.

**DESIGN AND SETTING:**

We conducted a systematic review and meta-analysis in accordance with the Preferred Reporting Items for Systematic Reviews and Meta-Analyses (PRISMA) guidelines in a Brazilian public university.

**METHODS:**

We sought to compile data from 6724 hospitalized patients with confirmed or suspected SARS-CoV-2 infection.

**RESULTS:**

Treatment with dexamethasone significantly reduced mortality within 28 days (risk ratio, RR: 0.89; 95% confidence interval, CI: 0.82-0.97). Dexamethasone use was linked with being discharged alive within 28 days (odds ratio, OR: 1.20; 95% CI: 1.07-1.33).

**CONCLUSIONS:**

This study suggests that dexamethasone may significantly improve the outcome among hospitalized patients with SARS-CoV-2 infection and associated severe respiratory complications. ­Further studies need to consider both dose-dependent administration and outcomes in early and later stages of the disease.

**PROSPERO platform:**

CRD42021229825.

## INTRODUCTION

By June 23, 2021, the coronavirus pandemic had reached 192 countries and regions with > 179 million and > 3.8 million confirmed cases and deaths, respectively.^1^ The Coronaviridae Study Group classified severe acute respiratory syndrome coronavirus 2 (SARS-CoV-2) within the family Coronaviridae, suborder Cornidovirineae, order Nidovirales.^2^

Given the disruption caused by lockdowns and social distancing, as well as overburdened healthcare systems, researchers are investigating different strategies for treating SARS-CoV-2 infections and are exploring drug repurposing.^3^ The World Health Organization Solidarity Trial report showed that treatments with drugs such as remdesivir, hydroxychloroquine, lopinavir and interferon had either little or no impact on mortality, need for intubation or overall hospital stay.^4^ On the other hand, corticosteroids (especially dexamethasone) might be beneficial for treating the SARS-CoV-2-induced cytokine storm.^5^ Data from the Recovery Trial noted that dexamethasone significantly reduced SARS-CoV-2-related deaths (by around 30% among patients receiving mechanical ventilation and by around 20% among those receiving oxygen alone).^6^ Therefore, several randomized controlled trials (RCTs) are underway to assess the effect of dexamethasone with regard to treatment of SARS-CoV-2 infection.

## OBJECTIVE

We sought to synthesize the evidence from randomized controlled trials (RCTs) on the clinical relevance of dexamethasone, compared with standard treatment, among hospitalized SARS-CoV-2 patients.

## METHODS

This study followed the guidelines for obtaining up-to-date and qualified biomedical literature.^7^ The review was registered on the PROSPERO platform (CRD42021229825). The inclusion criteria were that the studies needed to be RCTs that addressed hospitalized patients with confirmed or suspected ­SARS-CoV-2 infection, as reported in each study, either published or accepted for publication after the peer review. Other types of research designs, such as cross-sectional studies, as well as editorials, letters, reviews and study protocols, constituted exclusion criteria.

We independently searched the PubMed and Embase databases for RCTs. Publications were retrieved up to a cutoff date of February 14, 2021. The following MeSH terms were used: “coronavirus” OR “COVID-19” OR “2019-nCoV” OR “SARS-CoV-2” OR “severe acute respiratory syndrome” OR “SARS” AND “dexamethasone” AND “randomized controlled trial” or “randomized clinical trial”. Three experienced researchers (G.A., L.E.F. and J.P.X.) performed the searches and reviewed the abstracts. Each investigator independently selected studies for further inclusion, based on inclusion and exclusion criteria. The initial search returned 32 results from Embase and 193 from PubMed. After removing editorials, letters, reviews, protocols, duplicates and observational studies, two RCTs were found to fully satisfy our inclusion criteria (Appendix 1). These were found in Embase^8^^,^^9^ and PubMed.^9^ There were no disagreements between the review authors regarding the inclusion criteria. For each RCT, sample details, covariates analyzed, dosage and duration of dexamethasone treatment were extracted. The primary outcome measured was the risk ratio (RR) for death at 28 days and the secondary outcome was the odds ratio (OR) of being discharged alive within 28 days. In addition, these trials were assessed for potential risk of bias.^10^

## RESULTS

**Figure 1.**
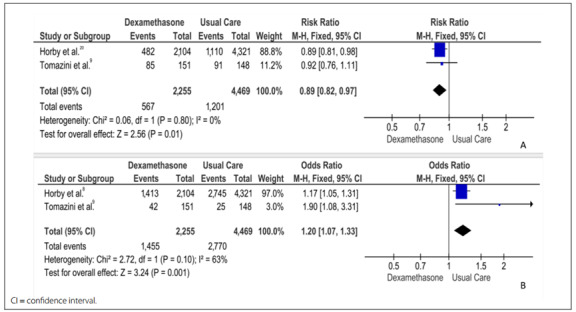
Risk ratio for dexamethasone with regard to mortality (A) and odds ratio for being discharged alive (B), among individuals with SARS-CoV-2 infection.

Data were extracted by D.S. and G.W. and were analyzed using RevMan 5.4.^11^ Through using the Cochrane Collaboration’s tool for assessing risk of bias in randomized trials, these two authors concluded that both of the RCTs included had low risk of bias, with the exception of the item “blinding of participants and staff”.^10^ To test the effect of dexamethasone on mortality, we obtained the RR from these two RCTs, which included 6724 patients. The results indicated that treatment with dexamethasone significantly reduced mortality within 28 days (RR = 0.89; 95% CI: 0.82, 0.97; I^2^ = 0%; Figure 1A). The effect of dexamethasone on the odds of being discharged alive within 28 days was significant (OR = 1.20; 95% CI: 1.07, 1.33; I^2^ = 63%; Figure 1B).^8^^,^^9^

Horby et al.^8^ published results from 6425 hospitalized patients (mean age = 66.1 ± 15.7 years; 36% female) who were admitted between March and June 2020 at 176 healthcare institutions in the United Kingdom with confirmed (~88%) or suspected SARS-CoV-2 infection (~10-12%). Most of the sample (56%) had preexisting diseases, among which at least one in five had diabetes, heart disease or chronic lung disease. Both the healthcare workers and the patients were aware of the treatment status. A 2:1 ratio with regard to receiving standard care (n = 4321) or standard care plus dexamethasone was adopted (n = 2104). Dexamethasone (6 mg) was administered orally or intravenously once daily for up to ten days. Mortality at 28 days was significantly lower in the intervention group (22.9%) than in the usual care group (25.7%). Deaths were lower in the interventions among patients receiving invasive mechanical ventilation (29.3% versus 41.4%) and among those receiving oxygen only (23.3% versus 26.2%). The effect of dexamethasone on mortality was not statistically significant among patients who did not receive any respiratory support at randomization. Hospitalization was shorter in the intervention group (median = 12 days) than in the usual care group (median = 13 days). The intervention group had a risk ratio of 1.10 with regard to being discharged alive within 28 days, and the largest effect in this regard was reported among patients who were receiving invasive mechanical ventilation at the time of randomization.^8^

Tomazini et al.^9^ reported results from 299 hospitalized patients with confirmed (> 95%) or suspected SARS-CoV-2 infection (mean age = 61 ± 14.0 years; 37% female). The most frequent underlying health condition was hypertension, in both groups (over 60%), followed by diabetes (over 37%). Recruitment take place between April and June 2020 at 41 intensive care units (ICUs) in Brazil. Both the healthcare workers and the patients were aware of the treatment status. A 1:1 ratio for receiving standard care (n = 148) or standard care plus dexamethasone was adopted. Dexamethasone was administered intravenously at a dosage of 20 mg daily for five days, and then 10 mg once daily for an additional five days or until discharge from the intensive care unit. In contrast to Horby et al.,^8^ the primary outcome was ventilator-free days within the first 28 days. The covariates included demographic and physiological data, history of corticosteroid use and time elapsed since symptom onset, among other related data. A higher number of ventilator-free days in the intervention group (6.6 days), in comparison with the standard treatment (4.0 days) within the first 28 days of treatment, was reported as the primary outcome. Mortality at 28 days was lower in the dexamethasone group (56.3%), compared with standard treatment (61.5%), although the differences were not statistically significant. In terms of length of hospital stay, the intervention group had an odds ratio (OR) of 1.90 of being discharged alive within 28 days (27.8% [dexamethasone] versus 16.9% [standard care]).^9^

## DISCUSSION

The results from this investigation showed that treatment with dexamethasone had a positive impact on mortality and length of hospitalization among SARS-CoV-2 hospitalized patients. However, the two RCTs assessed used different doses of the drug and both primary and secondary outcomes varied between the studies.^8^^,^^9^ Consequently, these data must be interpreted as preliminary at this stage and further RCTs are needed in order to obtain an accurate perspective regarding the role and regimen of dexamethasone for treating patients with mild, moderate and severe SARS-CoV-2 infection.

Currently, there are 64 clinical studies registered at ClinicalTrials.gov, which are at different phases and are being conducted in many regions of the world. Hopefully, these results could help immensely in clarifying the protocols that should be adopted for use of dexamethasone for treating SARS-CoV-2 infection.^15^

Although the safety of this drug has been demonstrated in relation to other infectious diseases, such as viral pneumonia,^16^^,^^17^ the risks from using corticosteroids among patients with mild to severe symptoms, including acute respiratory distress syndrome (ARDS) caused by SARS-CoV-2 infection,^18^^,^^19^ remain unclear.

In severe SARS-CoV-2 infection, the immunopathological responses appear to partially determine the outcome, which would explain why Horby et al.^8^ found specific effects in these cases. Early administration of glucocorticoids may impair antiviral activity. Another mechanism possibly related to shorter hospital stays and higher survival rates is the potential of dexamethasone for preventing pulmonary fibrosis in COVID-19 patients and in patients with ARDS that was not caused by SARS-CoV-2 (i.e. sepsis, trauma-induced shock and other viral infections).^13^^-^^14^

Importantly, the intervention group of Tomazini et al.^9^ was receiving concomitant treatment with other drugs such as hydroxychloroquine (23.8%) and azithromycin (69.9%), while Horby et al.^8^ adopted a 2:1 randomization methodology, thus raising some concerns regarding statistical power.^20^ Future studies will need to address these limitations through examining dose-dependent administration of dexamethasone in early and later stages of COVID-19 disease, taking into account the confounding effects of other medications and comorbidities.

## CONCLUSION

Through combining the evidence, healthcare professionals dealing with the current pandemic can be provided with relevant information, especially in countries where waves of SARS-CoV-2 infection are recurrent. This study suggests that use of dexamethasone could significantly improve the outcomes among critically ill patients with SARS-CoV-2 infection and associated severe respiratory complications.
